# Hypericin targets osteoclast and prevents breast cancer-induced bone metastasis via NFATc1 signaling pathway

**DOI:** 10.18632/oncotarget.22930

**Published:** 2017-12-04

**Authors:** Zhengxiao Ouyang, Xiaoning Guo, Xia Chen, Bo Liu, Qiang Zhang, Ziqing Yin, Zanjing Zhai, Xinhua Qu, Xuqiang Liu, Dan Peng, Yi Shen, Tang Liu, Qing Zhang

**Affiliations:** ^1^ Department of Orthopedics, The Second Xiangya Hospital, Central South University, Changsha, Hunan, P.R. China; ^2^ Department of Orthopedics, Shanghai Ninth People's Hospital, Shanghai Jiaotong University School of Medicine, Shanghai, P.R. China; ^3^ Department of Orthopedics, The First Affiliated Hospital of Nanchang University, Artificial Joints Engineering and Technology Research Center of Jiangxi Province, Nanchang, Jiangxi, P.R. China

**Keywords:** hypericin, osteoclast, bone metastasis, breast cancer, NFATc1

## Abstract

Bone is the most common target organ of metastasis of breast cancers. This produces considerable morbidity due to skeletal-related events, and severely reduces the quality of life. Increased osteoclast activity is implicated in breast cancer outgrowth in the bone microenvironment. Our previous observation of an anti-osteoclastic activity of hypericin, a natural plant compound, led us to investigate whether hypericin could inhibit bone metastasis and osteolysis caused by breast cancer. We find that hypericin inhibited the upregulation of osteoclasts stimulated by breast cancer cells. The activity of hypericin on osteoclasts and breast cancer-mediated osteoclastogenesis was associated with the inhibition of NFATc1 signaling pathway and attenuation of Ca^2+^ oscillation. Furthermore, hypericin suppresses invasion and migration in breast cancer cells, but has little effect on breast cancer-cell induced RANKL/OPG ratio in osteoblast or the expression of osteoclast-activating factors. Administration of hypericin could reduce tumor burden, osteolysis induced by direct inoculation of MDA-MB-231 cells into the bone marrow cavity of the tibia as well as metastasis of bone and improve survival in an experimental metastasis model by intracardiac injection of MDA-MB-231 breast cancer cells. Taken together, these results suggest that hypericin may be a potential natural agent for preventing and treating bone destruction in patients with bone metastasis due to breast cancer.

## INTRODUCTION

Currently, breast cancer is the most common female cancer worldwide and the second leading cause of cancer-related death in women [1, 2]. Bone metastasis is a debilitating aspect of breast cancer that occurs in 75 to 85% of women diagnosed with metastatic breast cancer [2, 3]. Bone metastases are frequently associated with complications, such as hypercalcemia because of osteolysis, nerve compression, intractable bone pain, and pathological fractures (also known as skeletal-related events [SREs]) [4]; these complications can result in morbidity and complex demands on the health care resources. In breast cancer bone metastases, although local bone-forming and osteoblastic lesions are observed, the dominant bone lesions are destructive and osteolytic [5]. Breast cancer cells secrete factors that act on the pre-osteoclasts, osteoblasts, and bone stromal cells to stimulate the production of mature osteoclasts, which degrade the bone, resulting in the release of growth factors that stimulate breast cancer cell proliferation and perpetuate a vicious osteolytic cycle [6]. Osteoclasts are the host cells responsible for bone resorption in such pathological situations. Thus, antiresorptive therapy, bisphosphonates (zoledronic acid), and the anti-receptor activator of nuclear factor kappa-B ligand (RANKL) antibody (denosumab) are standard of care to target osteoclast hyperactivity and prevent SREs [7].

However, the current treatments for bone metastasis have limited efficacy and are only palliative [8]. Side effects, such as renal toxicity and osteonecrosis of the jaw, are considered clinically significant, potentially painful, and debilitating enough to decrease the quality of life of patients with breast cancer [9]. In addition, clinical trials revealed that these bone-modifying agents did not significantly prolong the survival of the overall study population [9–11]. Thus, it is important to develop alternative therapeutic options to prevent bone metastasis and prolong the survival of patients with breast cancer bone metastasis.

Hypericin (HP), a naphtodianthrone, is one of the major active components of St. John's wort. It is shown to be clinically effective in treating depression [12] and human immunodeficiency virus (HIV) infection [13]. We previously proved that HP could suppress particle-induced osteolysis *via* modulation of the osteoclast function [14]. Recent evidence has suggested that HP exhibits cytotoxic activity against breast cancer cells [15, 16]. These previous findings collectively led us to hypothesize that treatment with HP might inhibit breast cancer-induced bone destruction. In the present study, we examined the effects of HP on osteolytic bone metastasis in mice subjected to intracardiac and intratibial injections of breast cancer cells. In addition, we investigated the molecular mechanisms underlying the inhibitory effects of HP against cancer cells and cancer-induced osteoclast differentiation *in vitro*.

## RESULTS

### Hypericin suppresses RANKL-induced osteoclastogenesis in an early stage

**Figure 1:**
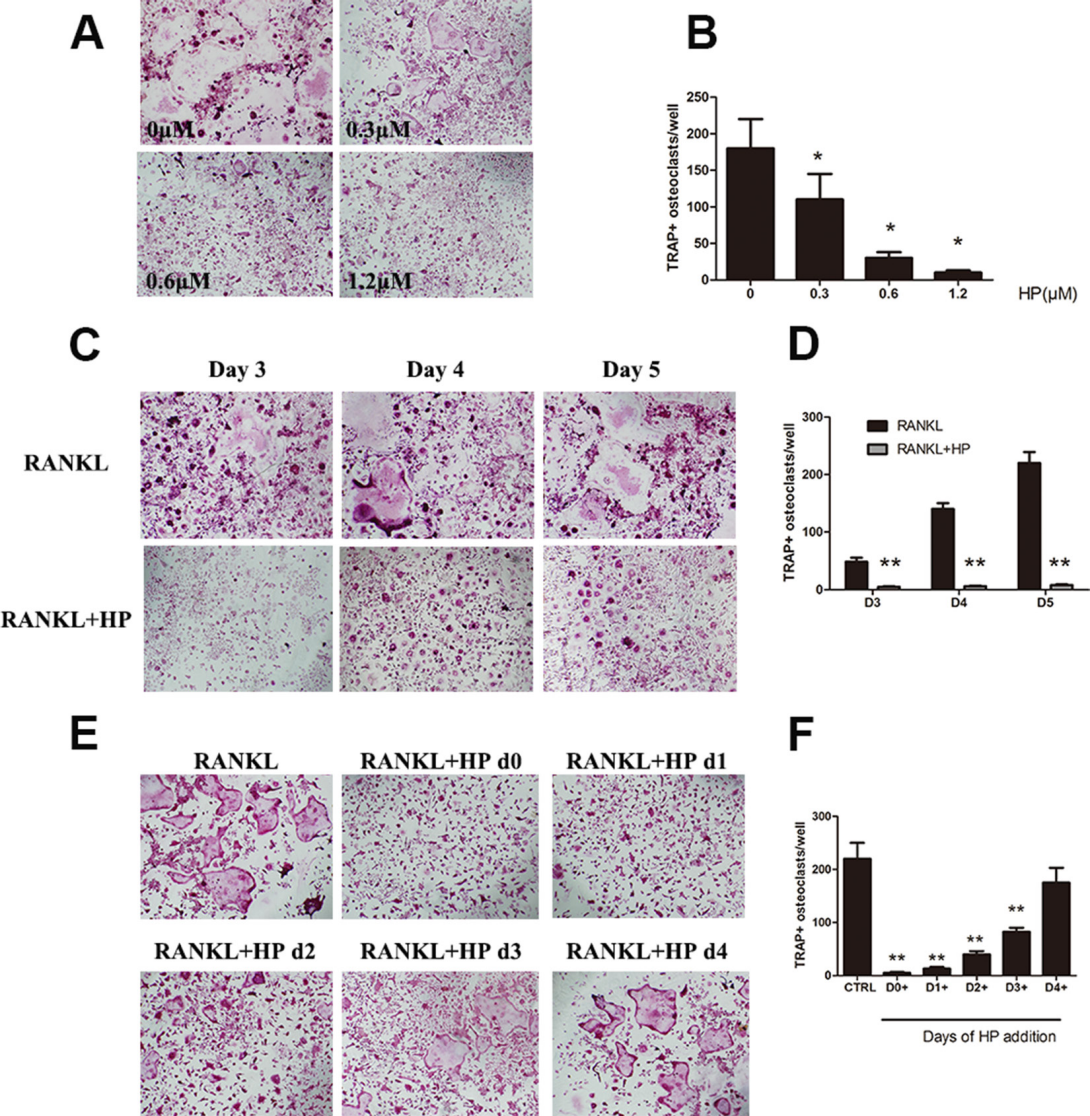
Hypericin suppresses RANKL-induced osteoclastogenesis (**A**) Effects of HP on RANKL-induced osteoclast differentiation. RAW264.7 cells (3 × 10^3^ cells/well) were stimulated with RANKL (50 ng/mL) or were untreated (controls), followed by treatment with the indicated doses of HP. After 5-7 days, cells were fixed and stained for measurement of TRAP expression. The cells were photographed (original magnification, 100×). (**B**) The TRAP-positive multinucleated (> 3 nuclei) osteoclasts were counted. Columns represent the mean results of experiments carried out in triplicate, whereas bars represent the standard deviation (SD). (**C**) RAW264.7 cells (3 × 10^3^ cells/well) were incubated in a medium supplemented with either RANKL (50 ng/mL) or RANKL and HP (1.2 μmol/L) for 3, 4, or 5 days and then stained for measurement of TRAP expression to examine osteoclast formation. TRAP-positive cells were photographed (original magnification, 100×). (**D**) The TRAP-positive multinucleated (> 3 nuclei) osteoclasts were counted. Columns represent the mean results of experiments carried out in triplicate, whereas bars represent the SD. (**E**) RAW264.7 cells (5 × 10^3^ cells/well) were incubated with RANKL (50 ng/mL), and then HP (1.2 μmol/L) was added on day 0, 1, 2, 3, or 4. After five days, cells were stained for measurement of TRAP expression. The cells were photographed (original magnification, 100×). (**F**) The TRAP-positive multinucleated osteoclasts were counted. Columns represent the mean results of experiments carried out in triplicate, whereas bars represent the SD.

RANKL induced the formation of numerous TRAP-positive multinucleated osteoclasts in the 0 μM group. However, the differentiation of osteoclasts was significantly inhibited after HP treatment, as evidenced by a dose-dependent decrease in the number of osteoclasts (Figure 1A, 1B). Moreover, TRAP-positive osteoclasts started to form after 3 days of RANKL stimulation. More mature osteoclasts were formed and fused over the following 2 days. In the HP treatment group, osteoclast differentiation was inhibited throughout the process (Figure 1C, 1D). In our previous study, HP was shown to exhibit no cytotoxic effects at the same doses that exerted inhibitory effects against osteoclast differentiation [14]. These data indicated that HP could suppress osteoclast formation in a dose-dependent manner.

To determine the stage at which HP inhibited osteoclastogenesis, HP (1.2 μM) was added to the culture medium on days 0, 1, 2, 3, or 4 of osteoclast differentiation. The maximum inhibitory effects of HP against osteoclastogenesis were observed when HP was added early with RANKL treatment (Figure 1E, 1F). Exposure of the precursor cells to HP at later stages (after 3 days) resulted in less effective suppression (Figure 1F, fifth column). These data suggested that HP could inhibit early osteoclast differentiation.

### Hypericin inhibits breast cancer-induced osteoclast differentiation and function via suppression of the NFATc1 signaling pathway and attenuation of Ca^2+^ oscillation

**Figure 2:**
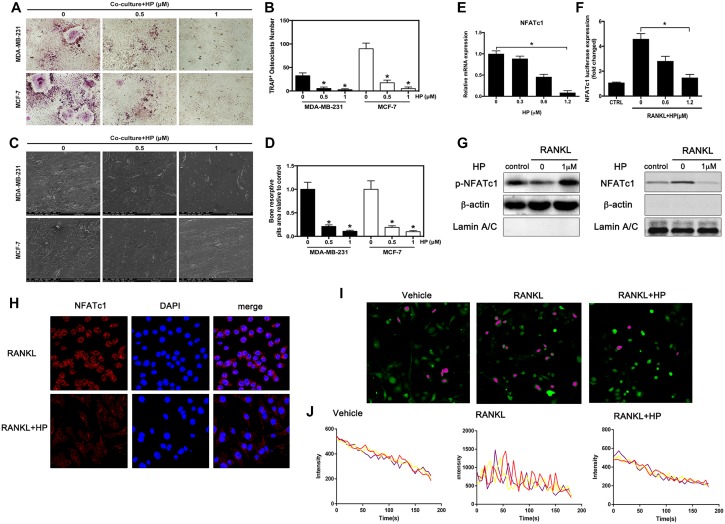
Hypericin inhibits breast cancer-induced osteoclast differentiation and function via suppression of the NFATc1 signaling pathway and attenuation of Ca^2+^ oscillation in osteoclasts (**A**) RAW264.7 cells (3 × 10^3^ cells/well) were incubated in the presence of MCF-7 or MDA-MB-231 cells for 24 h, exposed to HP (1 μmol/L) for 5 days, and finally stained for measurement of TRAP expression. (**B**) Multinucleated osteoclasts (> 3 nuclei) in co-cultures were counted. Columns represent the mean results of experiments carried out in triplicate, whereas bars represent the SD. (**C**) HP-inhibited osteoclast bone resorption induced by tumor cells. RAW264.7 cells (3 × 10^3^ cells/well) were seeded into bovine bone slices in the presence of MCF-7 or MDA-MB-231 cells for 24 h, and treated with HP (1 μmol/L) for 5 days. After 5 days of incubation, images were obtained using a scanning electron microscope (SEM). Images of bone resorption pits are shown. (**D**) Resorption pit areas were measured using ImageJ software. Columns represent the mean results of experiments carried out in triplicate, whereas bars represent the SD. (**E**) RAW264.7 cells were incubated in serum-free media containing the indicated concentrations of HP and RANKL for 24 h. The cells were lysed, and total RNA was subjected to RT-PCR for determination of NFATc1 gene expression. Graphs indicate the relative intensity of NFATc1 compared to that of GAPDH. (**F**) RAW264.7 cells that were stably transfected with a NFATc1 luciferase reporter construct were pretreated with the indicated concentrations of HP for 1 h and then incubated in the absence or presence of RANKL for 12 h. Luciferase activity was then determined using the Promega luciferase assay system. (**G**) RANKL-induced NFATc1 translocation to the nucleus was assessed by western blotting. RAW264.7 cells (1 × 10^6^ cells/well) were pretreated with the indicated concentrations of HP for 2 h and then stimulated with RANKL (50 ng/mL) or were untreated (controls) for 15 min. Cell nuclear extracts were prepared and subjected to western blotting using anti-NFATc1 and LaminA/C. Cell cytosol extracts were prepared and subjected to western blotting using anti-phospho-NFATc1 and actin. (**H**) Effects of HP on nuclear translocation of NFATc1 in RAW264.7 cells. Cells were treated with RANKL for 72 h in the presence and absence of HP (1 mmol/L) and stained with anti-NFATc1 antibody to investigate NFATc1 nuclear translocation (left panel). Nuclei were stained with DAPI (middle panel). Merged images of NFATc1 and the nuclei are shown in the right panel. (**I**) HP reduces intracellular Ca^2+^ levels and calcium influx. RAW264.7 cells (3 × 10^3^ cells/well) were incubated with RANKL (100 ng/mL) in the presence or absence of HP (1 μM) for 72 h. For Ca^2+^ measurement, cells were incubated with Fluo-4 AM and 0.05% pluronic F-127 (Invitrogen) in HBSS supplemented with 1% FCS and 1 mM probenecid (assay buffer) for 30 min followed by confocal analysis. Representative fluo-4 fluorescent images of the RAW264.7 cells from different treatment groups are shown. Pseudo-color-labeled (purple) area represents the cells that are actively undergoing fluorescence ratio changes. (**J**) The relative intracellular Ca^2+^ levels in individual cells were monitored for 5 min at 5-second intervals using the fluorescence intensity of Fluo-4 at 200× magnification. Cells with at least two oscillations were counted as oscillating cells. A minimum of 40 cells were monitored in triplicate wells. The average amplitude of Ca^2+^ oscillations in each cell was calculated using the TuneR and SeeWave packages for the R programming language. Representative traces of three randomly chosen BMMs were recorded in different treatment groups. The fluorescence ratio change was recorded every 5 s for 300 s.

Breast cancer cells can directly interact with the osteoclast precursor cells to stimulate osteoclast differentiation [17]. Considering the importance of the direct interaction between tumor cells and bone cells in osteoclast differentiation, we investigated the effects of HP on breast cancer-induced osteoclast differentiation in the co-culture system. In the co-culture system, MDA-MB-231 and MCF-7 cells markedly increased osteoclastogenesis (Figure 2A, 2B) and enhanced the osteoclastic bone resorption activity (Figure 2C, 2D) in the 0 μM group. These stimulatory effects of the breast cancer MDA-MB-231 cells were significantly reduced by HP. These results indicated that HP inhibited MDA-MB-231 and MCF-7 cell-mediated osteoclastogenesis and osteoclast activity. The molecular mechanism underlying the effects of HP on osteoclastogenesis was investigated. It has been reported that breast cancer cells stimulate osteoclastogenesis through direct or indirect RANKL secretion [18, 19]. Thus, we added RANKL to the culture medium to potentiate the stimulating effects of breast cancer cells. In response to the stimulatory effects of RANKL, osteoclast differentiation was shown to be associated with upregulation of the NFATc1 gene [20]. As shown by quantitative real-time PCR, RANKL dramatically induced the expression of NFATc1 in the 0 μM group. However, HP substantially suppressed NFATc1 expression in a dose-dependent manner (Figure 2E). Moreover, the results of the luciferase reporter assay further supported that NFATc1 activation was attenuated by HP (Figure 2F). At the protein level, addition of RANKL increased the phosphorylation of NFATc1 in the nucleus, which was associated with a decrease in plasma NFATc1 expression, showing that the NFATc1 signaling cascade was triggered during osteoclastogenesis; in addition, osteoclast inducer-mediated activation of NFATc1 signaling was inhibited by HP (Figure 2G, 2H). Moreover, we observed that RANKL stimulated the translocation of NFATc1 to the nucleus; which was inhibited by HP (Figure 2H).

Since Ca^2+^ oscillations are known as an important triggering signal for osteoclastogenesis [21], and Ca^2+^-NFATc1 signaling is an essential axis of osteoclast differentiation [20], we measured the change in intracellular Ca^2+^ concentration in RAW264.7 cells with or without RANKL treatment by Ca^2+^ imaging. RANKL markedly elevated the intracellular Ca^2+^ in RAW264.7 cells, as shown by measuring Fluo-3/AM fluorescence using confocal microscopy after 200 s (Figure 2I). Our previous results showed that HP could inhibit NFATc1 gene expression and phosphorylation; thus, we further tested the effects of HP on Ca^2+^ oscillation. Our results showed that HP treatment at 1 μM for 72 h dramatically reduced the Ca^2+^ oscillations in RANKL-stimulated cells (Figure 2J). The fluorescence intensities of five individual cells in each group were recorded within 200 s and statistically analyzed at a time point of 200 s. HP reversibly inhibited these RANKL-induced Ca^2+^ oscillations. Treatment with HP significantly suppressed the mean frequency of Ca^2+^ oscillations (Figure 2J). Our results indicated that HP could reduce both the average amplitude and frequency of RANKL-induced Ca^2+^ oscillations.

### HP has minimal effects on breast cancer cell-induced increase in RANKL/OPG ratio in osteoblasts

**Figure 3:**
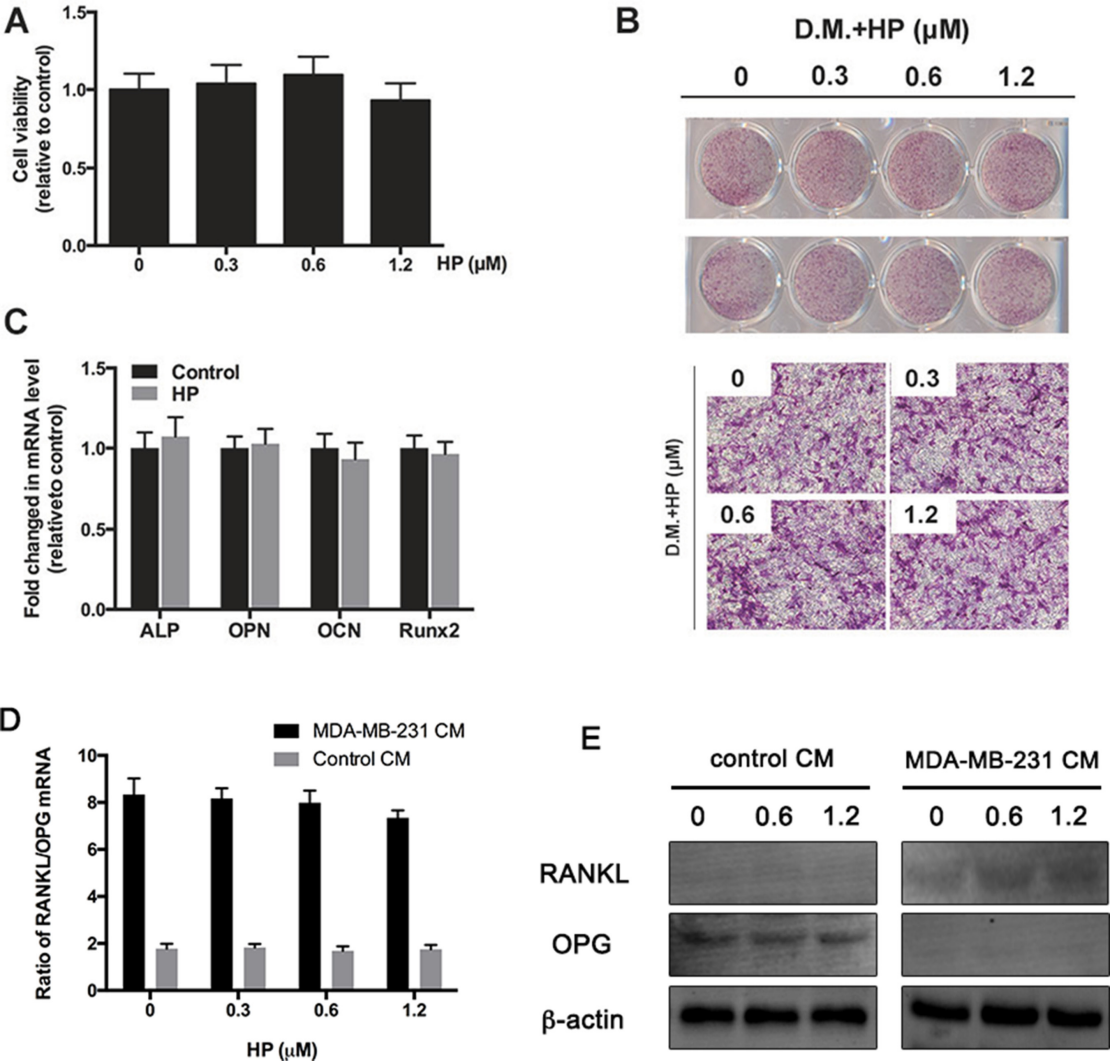
Hypericin has minimal effects on breast-cancer-cell induced increase in RANKL/OPG ratio in osteoblast (**A**) MC3T3-E1 cells were treated with the indicated concentrations of HP for 48 h. Cell viability was measured by the CCK-8 assay and is presented as percentages relative to the control group. (**B**) MC3T3-E1 cells were incubated in an osteogenic differentiation medium (DM) containing the indicated concentrations of HP. On day 14, differentiated cells were stained with ALP to detect the osteoblastic differentiation ability. Data represent the results of one of three experiments with similar results. (**C**) MC3T3-E1 cells were incubated with 1.2 μM HP for 7 days. The mRNA expression of ALP, OPN, OCN, and Runx2 genes was determined by real-time PCR and presented as ratios relative to the control group. All expression levels were normalized to GAPDH mRNA levels in corresponding samples. Data represent the means ± SD of three independent experiments (^*^P < 0.05). (**D**) Effects of HP on breast cancer cell-induced increase in RANKL/OPG ratio in osteoblasts. MDA-MB-231 cells (2 × 10^6^ cells/well) were treated with the indicated doses of HP for 24 h. A conditioned medium (CM) was harvested. In addition, bone marrow stromal cells (BMSCs; 1 × 10^5^ cells/well) were seeded onto 24-well plates and induced to differentiate *via* addition of ascorbic acid (50 g/mL), β-glycerophosphate (10 mM), and dexamethasone (10^−8^ M) for 7 days. Then, they were stimulated with CM for another 24 h. Total RNA was collected and subjected to quantitative real-time PCR using the indicated primers. (**E**) Effects of HP on breast cancer cell-induced RANKL and OPG protein expression level. MDA-MB-231 cells (2 × 10^6^ cells/well) were treated with the indicated doses of hypericin for 24 h. CM was harvested. Separately, BMSCs (1 × 10^5^ cells/well) were seeded onto 6-well plates and induced to differentiate *via* addition of ascorbic acid (50 g/mL), β-glycerophosphate (10 μM), and dexamethasone (10^−8^ M) for 7 days. Then, they were stimulated with CM for another 24 h. Whole cell extracts were prepared and subjected to western blot analysis as described in the materials and methods.

Osteoblasts are known to play a critical role in the deterioration of bone metastasis by increasing macrophage colony-stimulating factor (M-CSF) and RANKL expression and decreasing OPG expression [19]. The effects of HP on the secretion of M-CSF, RANKL, and OPG from the osteoblasts were assessed after breast cancer cell stimulation. To investigate the cytotoxic effects of HP on the osteoblasts, the viability of the mouse osteoblast cell line, MC3T3-E1 was tested. Hypericin exhibited no cytotoxic effects at the same dose that exerted inhibitory effects on the osteoclasts (Figure 3A). In addition, HP had minimal effects on both osteoblast differentiation (Figure 3B) and osteoblastic marker genes (ALP, OCN, OPN, and Runx2) at the same dose (Figure 3C). MDA-MB-231-CM increased RANKL expression and decreased OPG expression in the osteoblasts both at the mRNA and protein levels; however, HP did not affect the expression of RANKL and OPG in osteoblasts (Figure 3D, 3E). Our results indicated that HP exhibited minimal effects on the osteoblasts and breast cancer-induced increase in RANKL/OPG ratio in the osteoblasts.

### Hypericin suppresses the invasion and migration of breast cancer cells, but has minimal effects on the expression of osteoclast-activating factors

**Figure 4:**
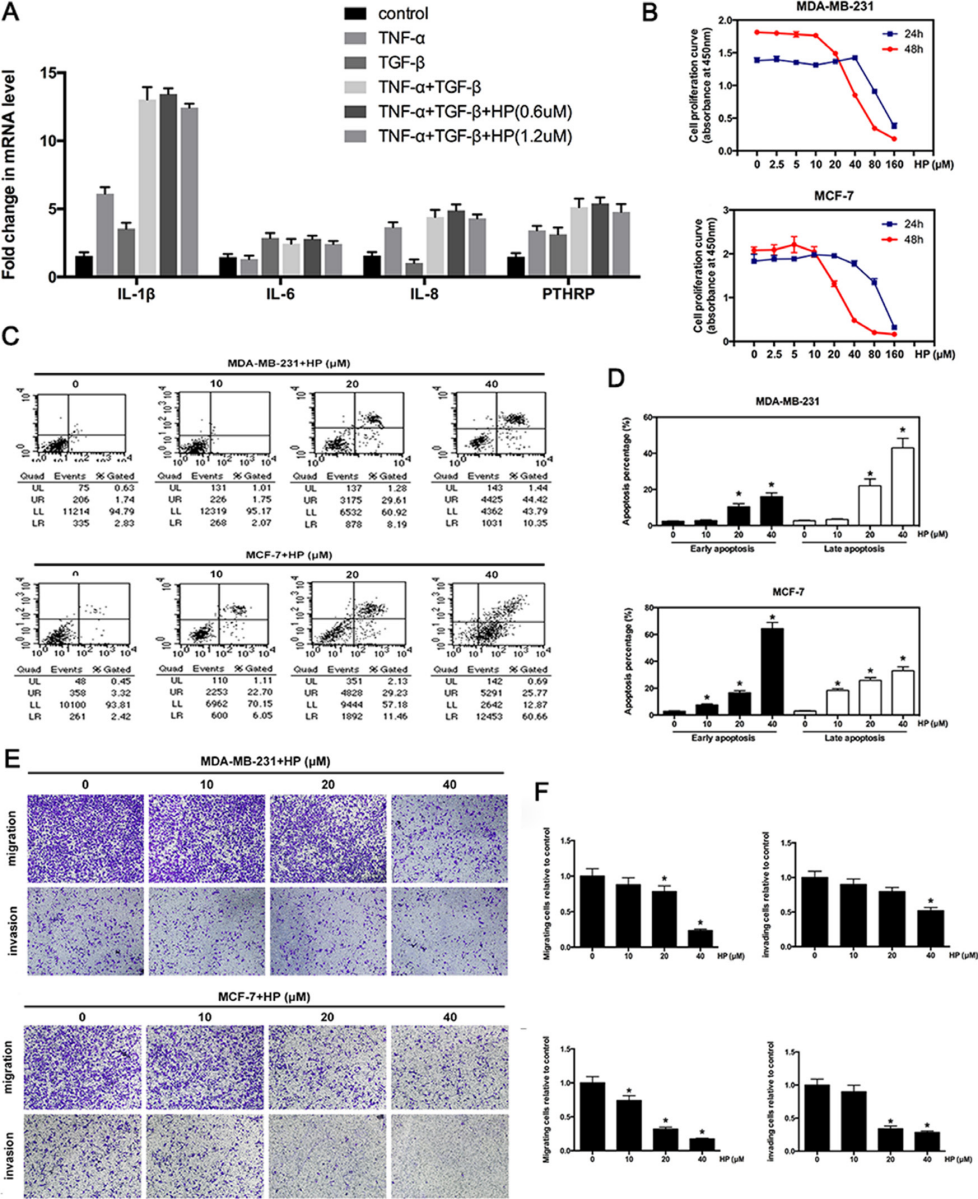
Hypericin inhibits the expression of osteoclast-activating factors and suppresses the invasion and migration of breast cancer cells (**A**) Effects of HP on the expression of osteoclast-activating factors induced by bone microenvironment cytokines. After pretreatment with the indicated concentrations of HP for 6 h, MDA-MB-231 cells (2 × 10^5^ cells/well) were stimulated with TNF-α (0.1 nM) or TGF-β (5 ng/mL), or TNF-α with TGF-β for 2 h. Total RNA was collected and subjected to quantitative real-time PCR using the indicated primers. (**B**) Effects of HP on the viability of breast cancer cells. MDA-MB-231 cells (8 × 10^3^ cells) and MCF-7 cells (8 × 10^3^ cells) were treated with the indicated concentrations of HP for 24 and 48 h. Cell viability was determined by the CCK8 assay. (**C**) HP promotes apoptosis at high concentrations. Flow cytometric analysis of HP-treated MDA-MB-231 and MCF-7 cells. (**D**) Percentage of apoptotic MDA-MB-231 and MCF-7 cells. Quantitative analysis of the percentage of apoptotic cells after 48-hour incubation. (**E**) Effects of HP on the inhibition of breast cancer cell migration and invasion. MDA-MD-231 and MCF-7 cells were starved for 12 h, and then seeded in the top chambers of transwells either with matrigel (for the invasion assay) or without matrigel (for the migration assay) in the presence of the indicated doses of HP. The bottom chambers of the transwells were filled with the a medium containing 10% FBS. Cancer cells were allowed to migrate for 6–8 h or invade for 10–12 h. The purple-stained cells, which migrated and invaded, showing irregular shape were photographed and counted. (**F**) Quantitative analysis of the percentage of cell migration and invasion using ImageJ. Columns represent the means of experiments performed in triplicate, whereas bars represent the SD.

In the bone microenvironment, cancer cells are exposed to growth factors, such as TGF-β, which are released from the degraded bone matrix [22]. These growth factors synergize with the proinflammatory factor, TNF-α to act on the cancer cells and accelerate the secretion of osteoclast-activating factors [23]. Because HP inhibited breast cancer-induced osteoclastogenesis, we next examined whether HP could also suppress the expression of osteoclast-activating factors in breast cancer cells. Our data showed that either TNF-α or TGF-β alone moderately promoted the expression of the osteoclast-activating factors (PTHrP, IL-1, IL-6, and IL-8). However, combined TNF-α and TGF-β were highly effective in stimulating osteoclast-activating factor expression (Figure 4A). However, HP only exerted little suppressive effect on the expression of osteoclast-activating factors.

To exclude the cytotoxic effects of HP on breast cancer-induced osteoclastogenesis, we next investigated the cytotoxic and apoptogenic effects of HP on breast cancer cells. Our results showed that HP did not exhibit cytotoxic or apoptogenic effects at the same doses that exerted inhibitory effects against osteoclast differentiation. However, HP exerted cytotoxic (Figure 4B) and apoptogenic (Figure 4C, 4D) effects at higher concentrations. Given the cytotoxic and apoptogenic effects of HP in breast cancer cells at high concentrations, we next examined its inhibitory effects against breast cancer migration and invasion, which are pivotal steps in cancer bone metastasis. Transwell assays showed that HP significantly suppressed the migration and invasion of two breast cancer cell lines (MDA-MB-231 and MCF-7) in a dose-dependent manner (Figure 4E, 4F).

### HP reduces osteolysis in human MDA-MB-231 breast cancer-bearing mice

**Figure 5:**
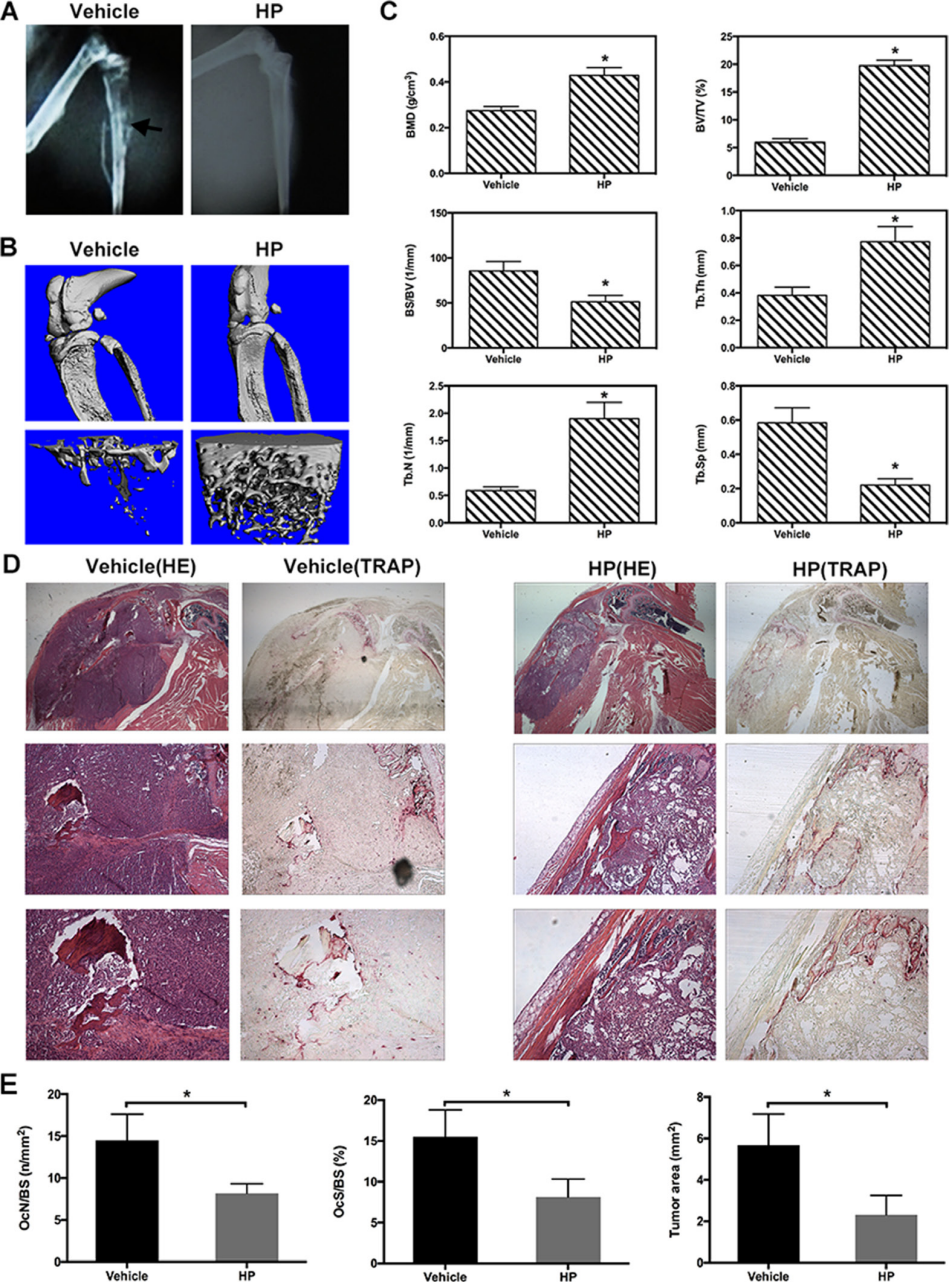
Hypericin reduces osteolysis and preserves trabecular/cancellous bone in human MDA-MB-231 breast cancer-bearing mice Female BALB/c nu/nu mice were intratibially injected with 1 × 10^7^ MDA-MB-231 cells/mL in PBS. From day 1 post-cell inoculation, animals were treated with the vehicle (PBS) or HP (9 mg/kg) 5 days/week until day 30. (**A**) Representative radiographs of the mice treated with the vehicle (left, upper) or HP (right, upper). Arrows indicate osteolytic bone lesions caused by injection of MDA-MB-231 cells. treatment with HP reduced these osteolytic bone lesions. (**B**) Three-dimensional computer reconstruction of the residual bone by micro-CT revealed extensive cancellous/trabecular bone loss in the vehicle-treated tumor-bearing mice (left, lower). HP inhibited bone loss in the tumor-bearing mice (right, lower). (**C**) Quantitative analysis of the bone parameters verified that MDA-MB-231 tumor cells induced osteolysis with a significant decrease in the BMD, BV/TV, Tb.Th, and Tb.N and increase in BS/BV and Tb.Sp. (**D**) Decalcified bones stained with H&E and TRAP. Vehicle-treated tumor-bearing mice exhibited serious osteolysis with discrete cortical bone. The tumor traversed the bone cortex and caused invasive metastasis outside the bone marrow cavity; some cells destroyed the metaphysis and gave rise to tumor growth in the articular cavity of the knee; TRAP-positive osteoclasts were observed alongside the junction zone between the tumor and bone tissues (left). HP treatment retained the intact bone cortex and complete metaphysis, and tumor tissues were localized in the bone marrow cavity; in addition, fewer TRAP-positive cells were observed in the HP-treated mice. (**E**) Quantitative analysis of the TRAP-positive osteoclasts using ImageJ software.

To determine whether HP could suppress osteolytic bone metastasis and osteolysis, nude mice were injected intratibially with human breast cancer MDA-MB-231 cells, which are triple negative (negative for estrogen receptor, progesterone receptor, and human epidermal growth factor receptor 2). Mice were then treated with HP (9 mg/kg/day) or the vehicle control (PBS) for 28 days. To examine the osteolytic bone metastasis, we performed microradiography, micro-CT, and histological examination. As shown in Figure 5A, osteolytic bone metastasis and destruction of cortices (arrows) were observed in the MDA-MB-231 tumor-bearing control mice (left, upper). In contrast, there were fewer osteolytic lesions, and the cortices remained intact in the HP-treated group (right, upper). Micro-CT confirmed that the vehicle-treated tumor-bearing mice showed extensive cancellous/trabecular bone loss in the tibia (Figure 5B). Quantitative analysis of the bone parameters showed that the MDA-MB-231 tumor-bearing control mice exhibited osteolysis, as evidenced by a significant decrease in BMD, BV/TV, Tb.Th, and Tb.N, and a significant increase in BS/BV and Tb.Sp (Figure 5C). However, HP reduced the extent of bone loss induced by MDA-MB-231 tumors, indicating that HP could protect against breast cancer-induced osteolysis. Histology further verified the protective effects of HP against MDA-MB-231 tumor-induced bone loss. As shown in Figure 5D, the vehicle-treated tumor-bearing mice exhibited serious osteolysis, resulting in discrete cortical bone and severe trabecular bone resorption, particularly alongside the tumor sites. Tumors traversed the bone cortex and invaded outside the bone marrow cavity, where some tumors destroyed the metaphysis, resulting in tumor growth in the articular cavity of the knee. HP treatment maintained the intact bone cortex and complete metaphysis, and tumor tissues remained in the bone marrow cavity. Bone histomorphometric analysis of the OcN/BS, OcS/BS, and tumor area in the proximal tibia further confirmed the protective effects of HP against osteolysis.

### HP reduces bone metastasis and increases survival in breast cancer-bearing mice

**Figure 6:**
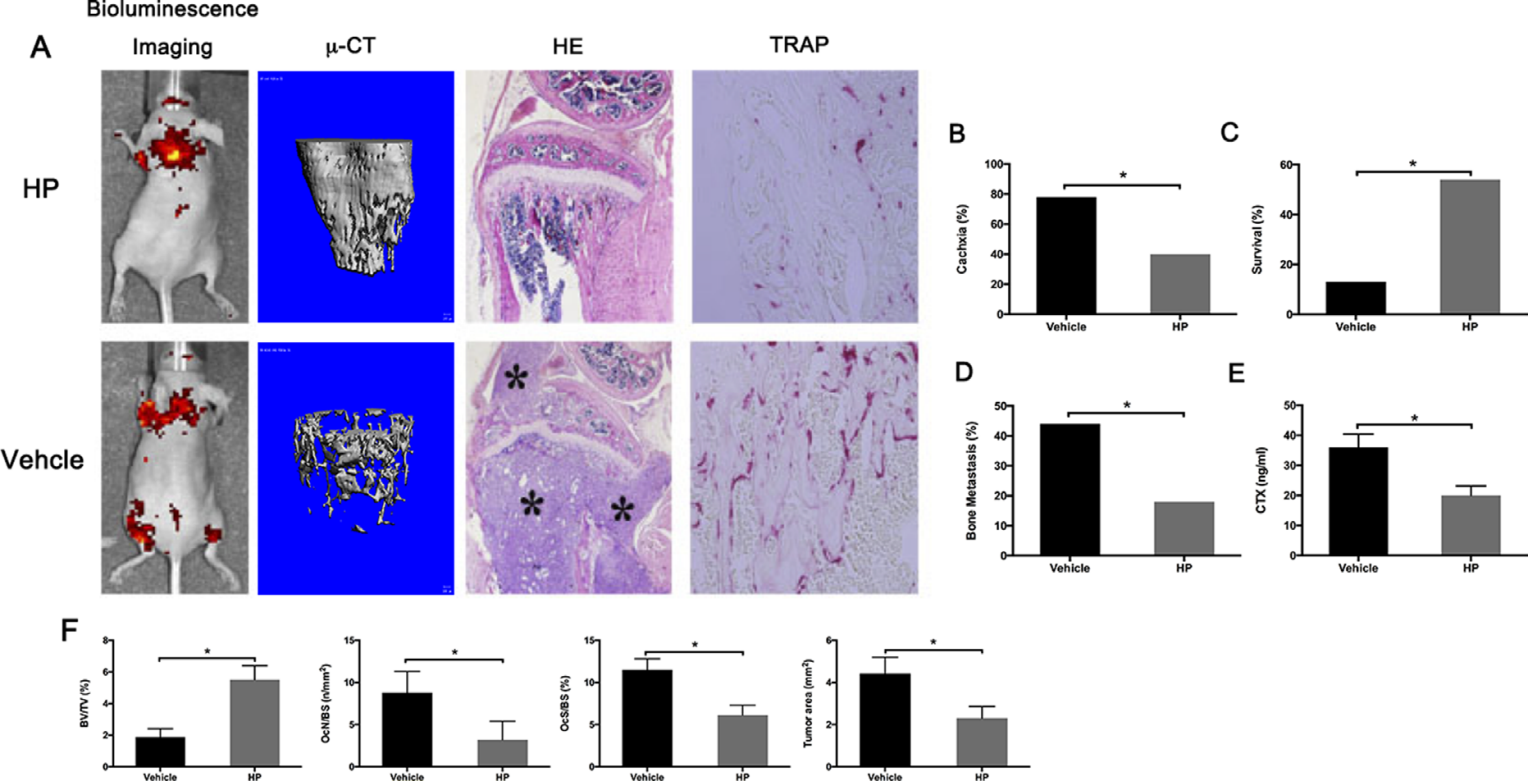
Hypericin reduces bone metastasis and increases survival in breast cancer-bearing mice Female BALB/c nu/nu mice were intratibially injected with 1 × 10^7^ MDA-MB-231 cells/mL stably transfected with luciferase (MDA-MB-231-LUC) in PBS. From day 1 post-cell inoculation, animals were treated with the vehicle (PBS) or HP (9 mg/kg/day) 5 days/week until day 30. (**A**) Representative live images, three-dimensional computer reconstruction of the residual bone by micro-CT, decalcified bones of the mice treated with the vehicle (down) or HP (upper) were stained with H&E and TRAP. Stars indicate osteolytic bone lesions caused by injection of MDA-MB-231-LUC cells. Treatment with HP reduced these osteolytic bone lesions. (**B**) Evaluation of cachexia (decrease in body weight). (**C**) Evaluation of survival. (**D**) Evaluation of bone metastasis incidence. (**E**) ELISA for determination of the serum levels of CTX. (**F**) Quantitative analysis of the bone parameters verified that MDA-MB-231-LUC tumor cells induced osteolysis with a significant reduction in the BV/TV. Histomorphometric evaluation of osteoclast number and surface/bone surface, and tumor area. ^*^P < 0.05 *versus* MDA-MB-231-LUC cell-injected mice.

In light of our findings and the inhibitory effects of HP against tumor migration, invasion, and proliferation, we hypothesized that HP might prevent bone metastasis. To test this hypothesis, we constructed an experimental metastasis model by intracardiac injection of MDA-MB-231 breast cancer cells, as previously described. In the vehicle-treated group, four of nine mice developed severe osteolytic bone lesions in the hind limbs 12 days after MDA-MB-231 injection, as determined by bioluminescence. However, only two of eight mice treated with HP developed osteolytic lesions. As shown by micro-CT, the osteolytic lesion area was significantly less in the HP-treated mice, compared to that in the vehicle-treated mice (Figure 6A, left). Micro-CT analysis confirmed that HP markedly reduced trabecular and cortical bone destruction induced by MDA-MB-231 injection (Figure 6F). Histomorphometric analysis of the osteolytic hind limbs showed that in the vehicle-treated mice, tumors filled the bone marrow cavity and eventually led to destruction of the cortical bone (asterisk). Administration of HP resulted in reduction of the tumor area. TRAP staining of the tibia sections showed that the number of osteoclasts at the tumor-bone interface decreased in the HP-treated mice, compared to that in the vehicle-treated mice (Figure 6A, right). In addition, injection of MDA-MB-231 cells resulted in a significant decrease in the survival and increase in cachexia, bone metastasis, and CTX, which was inhibited by HP administration (Figure 6B–6E). Our results indicated that HP could prevent bone metastasis of breast cancer and increase the survival.

## DISCUSSION

Approximately 80% of the patients with advanced breast cancer develop bone metastases. Once this occurs, it has historically been incurable, with survival from the time of the first diagnosis of skeletal involvement of approximately 24 months [9, 24]. Studies on the pathological processes that occur at the metastatic bone sites have led to the development of two effective drugs for treatment of bone metastasis; zoledronate (Zol), a third-generation bisphosphonate, and denosumab (Den), a fully human anti-RANKL antibody [1, 25]. Antiresorptive drugs have revolutionized the treatment and outcome in patients with bone metastases [26], where they decrease the incidence of skeletal complications and are now considered the standard of care for reducing SREs in patients with bone metastasis [5]. However, they have many side effects, including calcium homeostasis imbalance and hypocalcemia [5, 27]. Furthermore, 30–50% of the patients receiving such therapies still develop new bone metastasis and skeletal complications; in addition, clinical trials revealed that these bone-modifying agents did not significantly prolong the survival of the overall study population, emphasizing the need for new therapies [8–10]. We previously showed that HP could inhibit osteoclast differentiation [14] and have inhibitory effects on breast cancer [15, 16]. Thus, the purpose of this study was to investigate the effects of HP on breast cancer bone metastasis. To the best of our knowledge, this is the first study to demonstrate that HP could inhibit breast cancer-induced osteolytic bone metastasis *via* both decreasing the stimulatory effects of breast cancer on the osteoclasts and targeting breast cancer cells directly.

In the bone microenvironment, osteoclasts are the host cells responsible for bone resorption in both normal and pathological situations. Binding of RANKL to RANK is needed for osteoclast differentiation and is physiologically balanced by the secretion of OPG [28]. Once breast cancer cells metastasize to the bone, they drive the osteoclasts toward matrix resorption, which enhances the ratio of RANKL/OPG in the osteoblasts, and directly produce soluble mediators capable of inducing osteoclast maturation, such as soluble RANKL (sRANKL) [17, 29]. The unbalanced activation of osteoclasts results in massive bone resorption, which in turn induces the release of growth factors, such as TGF-β, from the bone matrix and promotes tumor growth that further increases the osteoclast activity [8, 26]. This feed-forward cycle creates a fertile microenvironment for tumor growth in the bone, which results in bone destruction and renders the tumor incurable. Thus, the primary goal in the management of bone metastases is to reduce the accelerated rate of bone destruction, thereby minimizing the skeletal complications. In the present study, we found that HP could inhibit tumor-induced osteoclastogenesis, resulting in a decrease in breast cancer-associated bone resorption in animal models. To gain insights into the molecular mechanism underlying HP-induced anti-osteoclastogenic effects, we investigated the RANKL/RANK signaling pathway, the primary pathway involved in osteoclast formation. Stimulation of RANK by RANKL has been reported to activate several signaling pathways, including phosphatidylinositol 3-kinase (PI3K)/AKT and mitogen-activated protein kinases (MAPKs), and subsequently activating a series of transcription factors, including nuclear factor kappa B (NF-κB) and NFATc1 [19]. Our previous findings showed that HP targeted the signaling pathway responsible for the activation of MAPK/ERK/c-fos [14]. In addition, it has been reported that c-fos expression is regulated by Ca^2+^/calmodulin-dependent protein kinases (CaMKs) [20]. Furthermore, Sorensen *et al.* have shown that HP acts as a potent protein kinase C (PKC) inhibitor, which inhibits osteoclastic acid secretion and bone resorption [30]. Intracellular Ca^2+^ puffs are involved in acid secretion that induces bone resorption [31]. Besides Ca^2+^, activation of PKC also requires phosphorylation as a cofactor [21]. NFATc1 is a Ca^2+^/calcineurin-dependent master regulator of osteoclastogenesis; moreover, Ca^2+^ oscillations and intracellular or extracellular Ca^2+^ release provide a digital Ca^2+^ signal that induces osteoclast upregulation and autoamplifies NFATc1 activation [21, 32]. Although the molecular basis of NFATc1 activation by Ca^2+^ oscillations has not yet been identified, immunoreceptor tyrosine-based activation motif (ITAM) and transient receptor potential cation channel, subfamily V (TRPV) might be involved [33]. Therefore, the Ca^2+^/NFATc1 signaling pathway is the most likely trigger of the effects of HP on osteoclastogenesis. Thus, we postulated that HP might disturb the regulation of Ca^2+^/NFATc1 in response to RANKL during osteoclastogenesis. Our findings showed that HP could decrease the intracellular Ca^2+^ concentration and oscillations and inhibit NFATc1 expression and translocation in RANKL-stimulated pre-osteoclastic RAW264.7 cells. It has been previously shown that Ca^2+^ oscillations in pre-osteoclasts are triggered by RANKL-dependent TRPV2 and store operated calcium entry (SOCE) activation or through inositol triphosphate (IP_3_) receptor activation and intracellular Ca^2+^ release [33]. However, the molecular basis is not fully elucidated and requires further investigation.

The estrogen receptor (ER) status of breast tumors is associated with a higher rate of both relapse to the bone and late-onset bone metastasis [18]. Thus, in this study, we compared the paracrine-induced osteoclastogenesis mediated by two human breast cancer cell lines; the estrogen-receptor positive MCF-7 cells and the estrogen-receptor negative MDA-MB-231 cells. Both cell lines exhibited a significant osteoclastogenic potential, as evidenced by a high TRAP activity and number of osteoclastic cells. In addition, differences in the osteoclastogenic potential between the cell lines were noted. Although MDA-MB-231 cells expressed higher RANKL levels [34], MCF-7 cell lines displayed a higher osteoclastogenic response, which might be potentially because MDA-MB-231 cells also expressed higher OPG levels. HP could suppress osteoclastogenesis induced in both cell lines. It has been reported that MCF-7 cells exhibit less RANKL expression; however, it still promote osteoclastogenesis, which could be partly inhibited by HP. There might be other mechanisms responsible for these inhibitory effects. Patients with estrogen receptor-positive breast cancer constitute a major clinical population who are at higher risk for bone metastases and have higher rate of both relapse to the bone and late-onset bone metastasis [35], potentially owing to the higher levels of RANKL expressed by MDA-MB-231 cells, compared to that by the MCF-7 cells [36]. Thus, we used MDA-MB-231 cells in the animal model.

Besides the osteoclasts, breast cancer cells can also interact with the osteoblasts to support osteoclast formation [22]. Breast cancer cells increase the expression of the osteoclast differentiation factors, M-CSF and RANKL, and decrease OPG levels in the osteoblasts, which could result in osteoclastogenesis [6]. Therefore, molecular targeting of cancer-mediated osteoclastogenesis or restoring the balance between the osteoblast-osteoclast interactions can reduce bone destruction. To investigate the effects of HP on the osteoblasts, we examined the proliferation and osteogenic ability of MC3T3E1 cells after HP treatment. HP did not significantly inhibit the growth of the osteoblasts or ALPase activity at a concentration of 1.2 μM, which inhibited the growth of RAW264.7 cells. Furthermore, RANKL/OPG ratio was also unaffected by HP treatment. The discrepancy in the effectiveness of HP could be attributed to the differences in the cell systems; in addition, RAW264.7 cells might be more sensitive to HP than osteoblasts.

The metastasis of breast cancer to the bone begins with the initiation of bone demolition, entry of malignant cells into the bone marrow, and a subsequent increase in osteolysis [6]. Tumor cells, which reach the bone microenvironment, secrete factors, such as PTHrP, which stimulate the osteoblasts to secrete RANKL, IL-8, IL-6, and IL-1. These factors increase the number of mature osteoclasts and activity of individual osteoclasts [5]. Understanding the molecular mechanisms that support and stimulate tumor osteolysis and regulate the survival might aid in the development of more effective therapies for this devastating complication of cancer. In our study, HP did not exert antiproliferative effects on breast cancer cells nor exert inhibitory effects on the stimulatory factors at low concentrations. However, when the concentration increased, we observed antiproliferative effects at 20 μM, which is consistent with the results of previous studies on the antitumor activity of HP in breast cancer cells [15]. In addition, inhibitory effects against the migration and invasion of breast cancer cells, which are the first steps for metastasis, were observed. Based on these findings and the clinical safety of HP [37], we used higher concentrations of HP in the animal model; however, this concentration is much lower than that required to induce antihyperalgesic effects [14].

In the *in vivo* study, we found that HP (9 mg/kg/day, intraperitoneally) decreased the tumor burden in the bone and osteolysis caused by direct intratibial injection of MDA-MB-231 cells. These results might be attributed to the inhibitory effects on the osteoclasts and tumor cells. Using the same HP concentration, we further confirmed that HP had a favorable tolerability and safety profile, as evidenced by the absence of visible side effects and organ toxicity in the mice (supplementary methods and results). Most studies have suggested that anti-osteoclast therapies can be effectively used to treat cancer-induced bone metastasis. However, it is still unknown whether they could prevent bone metastasis. The induction of bone metastasis in established models is achieved by direct injection of tumor cells into left ventricle [5, 17, 35, 38]. Thus, we constructed an animal model to test the preventive effects of HP. The results of the current study clearly showed that HP dramatically blocked spontaneous metastasis to bone in the spontaneous metastasis model. To the best of our knowledge, this is the first study on the functional consequences of HP treatment in mammary carcinoma *in vivo* ; in addition, it is the first study to show that HP could reduce bone metastasis. The preventive effects of HP might be owing to not only blocking cancer-induced osteoclastogenesis but also inhibiting the proliferation, invasion, and migration of breast cancer cells. It was previously shown that tumor cells localizing to the modified bone microenvironment appeared to preferentially localize in the osteoblast-rich areas [39], supporting that osteoblasts might be key components involved in providing the bone metastasis niche, and therefore a potential therapeutic target in breast cancer. Our data showed that HP exhibited minimal effects on the osteoblasts, which might also partly explain the preventive effects of HP. However, the inhibitory effects are not complete, which might be because other signaling pathways, such as LGR4, play a role in this process [40]. It acts as a second RANKL receptor, negatively regulating osteoclast differentiation and bone resorption.

At present, there are also many other treatments for breast cancer bone metastasis, such as microRNA [41, 42], the small-molecule antagonist adrenomedullin [24], combination therapy, such as OPG in combination with tamoxifen [35], LGR4 target therapy [40], interferon (IFN)-based therapies [43], and others. However, all of them are in the pre-clinical research stage, and potential adverse events need to be examined. Hypericin has been used in treatment of depression for many years; therefore, we can suppose that HP has potential in the treatment of breast cancer bone metastasis. Furthermore, its antidepressant and analgesic properties [44] would be beneficial to cancer patients. Bone metastasis constitutes a significant cause of morbidity and mortality in patients with many types of cancer, where it occurs in up to 70% of the patients with breast or prostate cancer and 15–30% of patients with lung, colon, stomach, bladder, uterus, rectum, thyroid, and kidney cancer [45]. The effects of HP on other tumor bone metastasis require further investigation.

Our study still has many limitations, particularly the regulation of tumor progression and metastasis by the surrounding microenvironment and the need for syngeneic *in vivo* models that allow investigation of the appropriate interactions. The immunodeficient mouse models might exclude the regulatory activity of the immune system on the metastatic process. Some researchers use the clinically relevant 4T1-derived syngeneic murine model of spontaneous mammary bone metastasis [17, 43]; however, there is a significant difference between the human and mouse cells, which cannot be ignored. The animal model used in this model reproducibly develops skeletal metastases [24, 46], which rarely colonize in other sites; therefore, it is valuable for our research. Many cytokines, such as ITGBL1 [47] and ET-1 [24], facilitate processes in the tumor cells, such as recruiting, residing, and growth in the bone and further stimulate osteoclast maturation in the bone microenvironment to form bone metastatic lesions. Moreover, many metastasis signature-associated genes are involved in bone-homing (CXCR4), angiogenesis (CTGF and FGF5), invasion (MMP-1 and ADAMTS1), and osteoclast recruitment (IL11) [48]. The ability of metastasis signature-associated genes to directly mediate metastasis remains unclear, which require further investigation to elucidate the effects of HP on such genes.

In conclusion, the results of this study showed that HP exhibited protective effects against breast cancer-induced bone destruction by directly decreasing the cancer cell-mediated osteoclast differentiation and bone resorption and inhibiting the invasion and migration of breast cancer. Taken together, it can be concluded that HP possesses dual ameliorating effects on breast cancer-associated osteolytic bone metastasis.

## MATERIALS AND METHODS

### Reagents and antibodies

Hypericin was purchased from Sigma-Aldrich (St. Louis, MO, USA). Alpha-minimum essential medium (MEM), fetal bovine serum (FBS), and penicillin were purchased from Gibico (Gaithersburg, MD, USA). Soluble mouse recombinant RANKL, human tumor necrosis factor-alpha (TNF-α), and transforming growth factor-beta (TGF-β) were purchased from R&D Systems (USA). Tartrate-resistant acid phosphatase (TRAP) staining solution was purchased from Sigma-Aldrich (USA). Primary antibodies against β-actin, phospho-nuclear factor of activated T-cells 1 (phospho-NFATc1), and NFATc1 were purchased from Cell Signaling Technology (USA). Cell counting kit-8 (CCK-8) was obtained from Dojindo Molecular Technologies (Dojindo, Japan).

### Cell lines

RAW264.7, MCF-7, MDA-MB-231, and MC3T3-E1 cells were obtained from the American Type Culture Collection. The luciferase-labeled human breast cancer cell line (MDA-MB-231-LUC-D3H2LN) was purchased from Caliper Life Science Inc, and cultured in Dulbecco's modified Eagle's medium (DMEM) containing 10% FBS. RAW264.7 cells were cultured in alpha-MEM supplemented with 10% FBS and antibiotics. This cell line is a well-established osteoprogenitor cell system that expresses RANK and differentiates into functional TRAP-positive osteoclasts when cultured with soluble RANKL [49]. MDA-MB-231, MCF7, and MC3T3-E1 cells were cultured in alpha-MEM supplemented with 10% FBS and antibiotics.

### *In vitro* osteoclastogenesis assay

RAW264.7 cells were cultured in 96-well plates at a density of 3 × 10^3^ cells/well and allowed to adhere overnight. The medium was replaced, and the cells were treated with 50 ng/mL RANKL for 7 days. All cell lines were stained with TRAP using a leukocyte acid phosphatase kit. For co-culture experiments with tumor cells, RAW264.7 cells were seeded at 3 × 10^3^ cells/well and allowed to adhere overnight. The following day, MDA-MB-231 and MCF-7 cells at 1 × 10^3^ cells/well were added to the RAW264.7 cells, treated with HP, and co-cultured for 7 days before TRAP staining. TRAP-positive multinucleated cells with > five nuclei were counted as osteoclasts.

### Bone absorption assay

RAW264.7 cells were seeded at a density of 3 × 10^3^ cells/well onto bovine bone slices. The experiment was carried out in triplicate. The following day, MDA-MB-231 and MCF-7 cells at 1 × 10^3^ cells/well were added to the RAW264.7 cells, treated with HP, and co-cultured for 7 days before TRAP staining. Cells adhering to the bone slices were then removed by mechanical agitation and sonication. Resorption pits were visualized using a scanning electron microscope (SEM; FEI Quanta 250), and the bone resorption area was quantified using ImageJ software (National Institutes of Health, Bethesda, MD, USA).

### *In vitro* osteoblast differentiation assay

MC3T3-E1 cells were incubated in 24-well plates at 5 × 10^3^ cells/well and allowed to adhere overnight. The medium was replaced, and the cells were treated with osteogenic differentiation medium and different concentrations of HP for 14 days. All cell lines were stained with alkaline phosphatase (ALP) assay kit (Abcam, USA).

### Immunocytochemistry

Cells cultured on glass coverslips were washed with phosphate-buffered saline (PBS), fixed using 4% paraformaldehyde in PBS for 10 min, and then permeabilized with 0.05% Triton-X 100 in PBS for 5 min. Fixed cells were incubated with rabbit anti-NFATc1 antibody (Gene Tex, Inc., Irvine, CA; dilution, 1:200 in PBS) at 4°C overnight after blocking of the nonspecific binding sites with 5% normal goat serum in PBS for 30 min at room temperature. After washing with PBS, the antibody was visualized by incubation with Alexa Fluor 488-conjugated goat anti-rabbit IgG secondary antibody (1 μg/mL; Molecular Probes, Eugene, OR) for 30 min at room temperature. Nuclear staining was performed using 4,6-diamidino-2-phenylindole (DAPI) dye (Dojindo, Kumamoto, Japan).

### Real-time PCR analysis

For real-time quantitative polymerase chain reaction (PCR) analysis, total cellular RNA was extracted from the cells using TRIzol reagent (Invitrogen, Carlsbad, CA, U.S.), as previously described [36]. PCR primers were as follows:

### Mouse ALP

5′-GCTGGAAACCATGATCACCT-3′ (forward); 5′-GAGTTGCCACACAGCATCAC-3′ (reverse); Mouse osteocalcin (OCN); 5′-CAGACACCATGAGGA CCATC-3′ (forward); 5′-GGACTGAGGCTCTGTG AGGT-3′ (reverse); Mouse osteopontin (OPN); 5′-CTTTCACTCCAATCGTCCCTA-3′ (forward); 5′-GCTCTCTTTGGAATGCTCAAGT-3′ (reverse); Mouse runt-related transcription factor 2 (Runx2); 5′-AGGGACTATGGCGTCAAACA-3′ (forward); 5′-GGCTCACGTCGCTCATCTT-3′ (forward); Mouse osteoprotegerin (OPG); 5′-CAGATGGGTT CTTCTCAGGT-3′ (forward); 5′-TCTCGGCATT CACTTTGGTC-3′ (reverse); Mouse RANKL ; 5′-ATCCCATCGGGTTCCCATAA-3′ (forward); 5′-TTCG TGCTCCCTCCTTTCAT-3′ (reverse); Mouse NFATc1; 5′-TGGAGAAGCAGAGCACAGAC-3′ (forward); 5′-GCGGAAAGGTGGTATCTCAA -3′ (reverse); Human interleukin (IL)-1; 5′-AATCTCCGAC CACCACTACA-3′ (forward); 5′-CATATGGACCAGA CATCACC-3′ (reverse); Human IL-6 ; 5′-AAGAGG CACTGGCAGAAAAC-3′ (forward); 5′-TTGGGTCA GGGGTGGTTATT-3′ (reverse); Human IL-8 ; 5′-TTGGCAGCCTTCCTGATTTC-3′ (forward); 5′-AAC TTCTCCACAACCCTCTG-3′ (reverse); Human parathyroid hormone-related protein (PTHrP) ; 5′-CGACGATTCTTCCTTCACCA-3′ (forward); 5′-TTTCCTGCTCCTTGCGTTTC-3′ (reverse); Mouse β-actin ; 5′-GTACGCCAACACAGTGCTG-3′ (forward); 5′-CGTCATACTCCTGCTTGCTG-3′ (reverse); Human glyceraldehyde 3-phosphate dehydrogenase (GAPDH); 5′-GGGCATCTTGGGCTACACT-3′ (forward); 5′-GCCGAGTTGGGATAGGG-3′ (reverse).

### Western blotting

Cells were lysed in radioimmunoprecipitation assay (RIPA) lysis buffer containing 50 mM Tris–HCl, 150 mM NaCl, 5 mM ethylenediaminetetraacetic acid (EDTA), 1% Triton X-100, 1 mM NaF, 1 mM sodium vanadate, and 1% deoxycholate and protease inhibitor cocktail. The lysate was centrifuged at 12,000 rpm for 10 min, and the protein in the supernatant was collected and quantified. Each protein lysate (30 μg) was resolved using 8–10% sodium dodecyl sulfate-polyacrylamide gel electrophoresis (SDS-PAGE) and transferred to a polyvinylidene difluoride membrane (Millipore, Bedford, MA, USA). Nonspecific interactions were blocked with 5% skim milk for 2 h. The membranes were probed with primary antibodies overnight at 4°C. They were then incubated with the appropriate horseradish peroxidase-conjugated secondary antibodies, and the reactivity was detected using an odyssey infrared imaging system (LI-COR).

### Luciferase reporter gene activity assay

The effects of HP on RANKL-induced NFATc-1 activation were measured in RAW264.7 cells that were stably transfected with an NFATc-1 luciferase reporter construct, as previously described [14, 50]. Briefly, cells were seeded into 48-well plates (three replicates) and maintained in a cell culture medium for 24 h. Cells were then incubated in the presence or absence of the indicated concentrations of HP for 1 h, followed by incubation with RANKL (50 ng/mL) for 8 h. Luciferase activity was measured using a luciferase assay system (Promega, Madison, WI, USA) and normalized to that of the vehicle control.

### Intracellular Ca*^2+^* oscillation measurement

RAW264.7 cells (4 × 10^3^ cells/well) were seeded into 96-well plates, treated with supplements based on the assigned groups, and cultured for 72 h. Cells in the treatment groups were treated with RANKL (50 ng/mL) and HP (1.2 μM); cells in the positive control group were treated with RANKL (50 ng/mL) only; and cells in the control group were treated with α-MEM only. After 72 h, the cells were incubated in 5 μM Fluo-4 AM and 0.05% pluronic F-127 (Invitrogen) in Hank's balanced salt solution (HBSS) supplemented with 1% fetal calf serum (FCS) and 1 mM probenecid (assay buffer) for 30 min at 37°C. Cells were washed twice with the assay buffer and incubated at room temperature for 20 min. Cells were excited at 488 nm using an inverted fluorescence microscope (Olympus). The relative intracellular calcium levels in individual cells were monitored for 5 min at 5-second intervals based on the fluorescence intensity of Fluo-4 (200× magnification). Cells with at least two oscillations were counted as oscillating cells. A minimum of 40 cells were monitored in triplicate wells. The average amplitude of calcium oscillation in each cell was calculated using the TuneR and SeeWave packages for the R programming language [51].

### Cytotoxicity assay

The effects of HP on MDA-MB-231, MCF-7, and MC3T3E1 cell proliferation were determined using the CCK-8 (Dojindo Molecular Technology, Japan). Cells were plated in 96-well plates at 3 × 10^3^ cells/well in triplicate. After 24 h, the cells were treated with increasing concentrations of HP for 48 h or other indicated times. Then, 10 μL of CCK-8 was added to each well, and the plates were incubated at 37°C for 2 h. The optical density (OD) was measured using an ELX800 absorbance microplate reader (Bio-Tek, USA) at 450 nm (650 nm reference). Cell viability was calculated relative to the control, as follows: cell viability = [experimental group OD - blank OD]/[control group OD-blank OD].

### Apoptosis assay

The apoptotic effects of HP on MDA-MB-231 and MCF-7 cells were determined using the Vybrant apoptosis assay kit #2 (Invitrogen, USA). Cells were treated with increasing concentrations of HP for 48 h. Then, they were washed twice with cold PBS and pelleted; the supernatants were discarded, and the cells resuspended in annexin binding buffer. Early apoptosis was detected by staining with Alexa Fluor 488 annexin V and propidium iodide. Fluorescence-activated cell sorting (FACS) was performed using a FACScan flow cytometer (Becton-Dickinson, Sunnyvale, CA, USA). Data were acquired using CELL Quest software.

### Transwell migration and invasion assay

The transwell assay was performed as previously reported [52]. Briefly, transwell membrane was used for the migration assay. A membrane coated with matrigel (100 g/mL, 100 L/well) was used for the invasion assay. The top chambers were seeded with MCF-7 or MDA-MB-231 cells at 3 × 10^3^ cells/well with different concentrations of HP (0, 10, 20, and 40 μM). The bottom chambers were filled with 500 μL of the medium supplemented with 10% FBS. After 8-hour incubation (for migration) or 12-hour incubation (for invasion), the migrated cells were fixed with 4% paraformaldehyde and stained with 1% crystal violet. Images were acquired using an Olympus inverted microscope, and the migratory cells were counted using Image-Pro Plus 6.0.

### *In vivo* osteolytic bone metastasis

All animal procedures and experimental protocols were approved by the Institutional Animal Care and Use Committee of Central South University. Cultured human breast cancer MDA-MB-231 cell line was resuspended in PBS at a final concentration of 1 × 10^7^ cells/mL. BALB/c nu/nu mice (5-6 weeks old; female; Harlan) were inoculated with MDA-MB-231 cells (l × 10^7^ cells/mL) directly into the tibiae plateau using a percutaneous approach. The mice were randomly assigned to one of two groups; vehicle-treated (0.9% sodium chloride, *n* = 8) or HP-treated group (9 mg/kg/day, *n* = 8). Hypericin was administered intraperitoneally for 28 days, and then the mice were sacrificed. Radiographs (Faxitron Radiographic inspection unit; Kodak) were obtained at the baseline and just prior to sacrifice. The tibiae of all animals were scanned by micro-computed tomography (micro-CT; Skyscan 1072; Skyscan, Aartse-laar, Belgium). Bone histomorphometric analyses were performed based on the micro-CT data, as previously described [14]. The bone mineral density (BMD), as well as the microstructural indices of trabecular bone density (BV/TV), bone surface/volume ratio (BS/BV), trabecular thickness (Tb.Th), trabecular number (Tb.N), and trabecular space (Tb.Sp) were measured to assess the bone microstructure of the tibiae. Tissues were removed, fixed with 4% paraformaldehyde (Sigma-Aldrich) for 1 day at 4°C, and decalcified using 12% EDTA. Decalcified bones were embedded in paraffin and sectioned. For histological examination, sections were stained with hematoxylin and eosin (H&E), and other sections were stained with TRAP to identify osteoclasts on the bone surface. Bone static histomorphometric analyses of the osteoclast surface (the percent of trabecular bone surface covered by osteoclast, OcS/BS), osteoclast number per bone surface (OcN/BS), and tumor area were performed using a professional image analysis software (ImageJ, NIH, USA) under a microscope (Leica image analysis system, Q500MC).

For experimental spontaneous bone metastasis studies, 1 × 10^6^ MDA-MB-231-GFP cells were injected into the left cardiac ventricle of BALB/c nu/nu mice (5-6 weeks old; female; Harlan), as previously described [41]. Development of primary tumors was analyzed by bioluminescence imaging using a Xenogen IVIS 200 imaging system (Caliper Life Sciences) at week 4. At this time point, the mice were sacrificed, and the osteolytic lesions were observed using the SkyScan micro-CT system. In addition, bone metastasis was evaluated by H&E staining. TRAP staining was performed to identify the mature osteoclasts in the metastatic bone lesions. Enzyme-linked immunosorbent assays (ELISA) were performed to determine the levels of type I collagen cross-linked C-telopeptide (CTX) in the blood. The number of TRAP-positive multinucleated osteoclasts normalized with the bone area (OcN/BA) and percentage of osteoclast area per bone area (OcA/BA, %) were assessed in each sample.

### Statistical analysis

All data are presented as the mean ± standard deviation (SD) of the values obtained from three or more experiments. Statistical significance was tested using paired one-way analysis of variance (ANOVA), where appropriate. *P*-values < 0.05 were considered statistically significant.

## SUPPLEMENTARY MATERIALS FIGURES


