# Correction: Hypericin targets osteoclast and prevents breast cancer-induced bone metastasis via NFATc1 signaling pathway

**DOI:** 10.18632/oncotarget.27273

**Published:** 2019-10-22

**Authors:** Zhengxiao Ouyang, Xiaoning Guo, Xia Chen, Bo Liu, Qiang Zhang, Ziqing Yin, Zanjing Zhai, Xinhua Qu, Xuqiang Liu, Dan Peng, Yi Shen, Tang Liu, Qing Zhang

**Affiliations:** ^1^ Department of Orthopedics, The Second Xiangya Hospital, Central South University, Changsha, Hunan, P.R. China; ^2^ Department of Orthopedics, Shanghai Ninth People’s Hospital, Shanghai Jiaotong University School of Medicine, Shanghai, P.R. China; ^3^ Department of Orthopedics, The First Affiliated Hospital of Nanchang University, Artificial Joints Engineering and Technology Research Center of Jiangxi Province, Nanchang, Jiangxi, P.R. China

**This article has been corrected: **Due to errors in image processing, the representative image of TRAP staining in RANKL+HP d1 is incorrect. The proper Figure 1 is shown below. The authors declare that these corrections do not change the results or conclusions of this paper.

Original article: Oncotarget. 2018; 9:1868–1884. 1868-1884. https://doi.org/10.18632/oncotarget.22930

**Figure 1:**
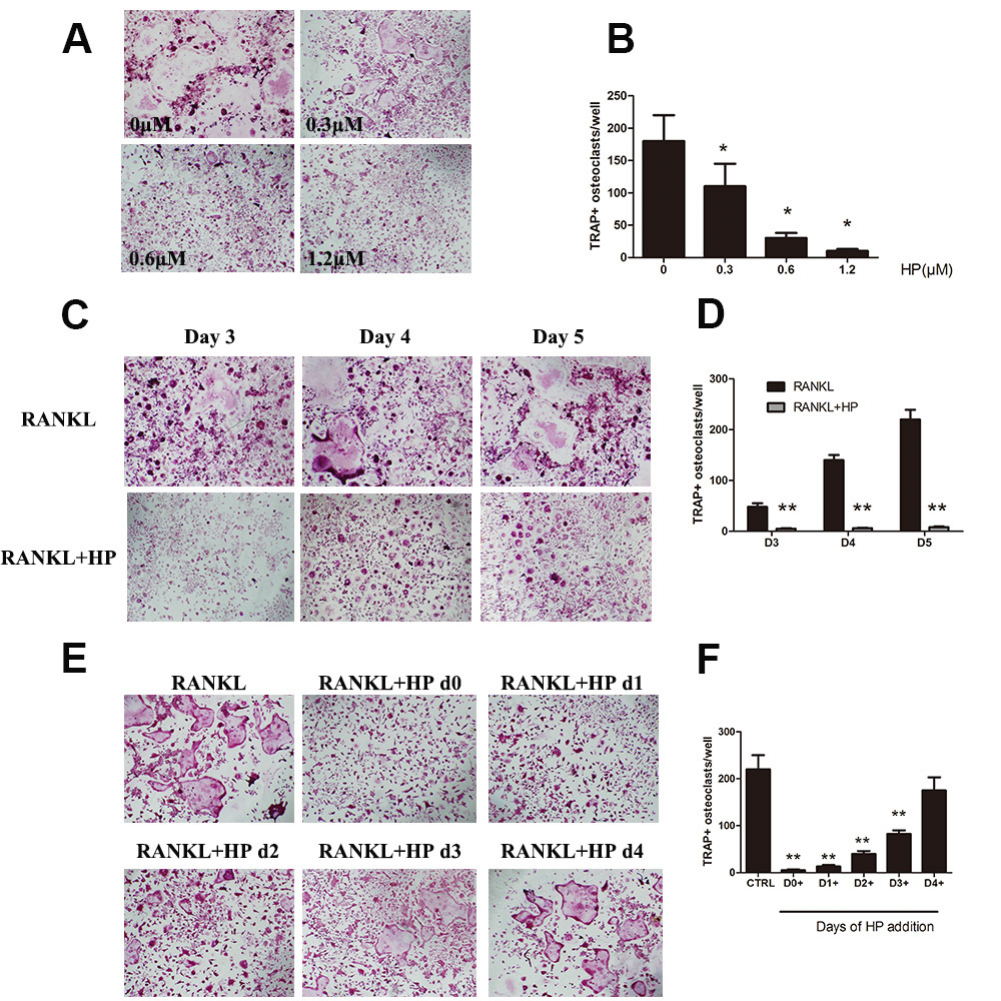
Hypericin suppresses RANKL-induced osteoclastogenesis. (**A**) Effects of HP on RANKL-induced osteoclast differentiation. RAW264.7 cells (3 × 10^3^ cells/well) were stimulated with RANKL (50 ng/mL) or were untreated (controls), followed by treatment with the indicated doses of HP. After 5-7 days, cells were fixed and stained for measurement of TRAP expression. The cells were photographed (original magnification, 100×). (**B**) The TRAP-positive multinucleated (> 3 nuclei) osteoclasts were counted. Columns represent the mean results of experiments carried out in triplicate, whereas bars represent the standard deviation (SD). (**C**) RAW264.7 cells (3 × 10^3^ cells/well) were incubated in a medium supplemented with either RANKL (50 ng/mL) or RANKL and HP (1.2 μmol/L) for 3, 4, or 5 days and then stained for measurement of TRAP expression to examine osteoclast formation. TRAP-positive cells were photographed (original magnification, 100×). (**D**) The TRAP-positive multinucleated (> 3 nuclei) osteoclasts were counted. Columns represent the mean results of experiments carried out in triplicate, whereas bars represent the SD. (**E**) RAW264.7 cells (5 × 10^3^ cells/well) were incubated with RANKL (50 ng/mL), and then HP (1.2 μmol/L) was added on day 0, 1, 2, 3, or 4. After five days, cells were stained for measurement of TRAP expression. The cells were photographed (original magnification, 100×). (**F**) The TRAP-positive multinucleated osteoclasts were counted. Columns represent the mean results of experiments carried out in triplicate, whereas bars represent the SD.

