# Supplementary material for: Hypericin targets osteoclast and prevents breast cancer-induced bone metastasis via NFATc1 signaling pathway
Source: Oncotarget. 2017 Dec 4;9(2):1868–84. doi: 10.18632/oncotarget.22930 (PMC5788605; doi:10.18632/oncotarget.22930)
Supplement: Supplementary file 1 [file oncotarget-09-1868-s001.pdf]

Hypericin targets osteoclast and prevents breast cancer-induced bone metastasis via NFATc1 signaling pathway

SUPPLEMENTARY MATERIALS

MATERIALS AND METHODS

In vivo bio-safety evaluation

After scarified, blood samples were taken from animals from each group. Then blood biochemical tests (liver, heart and renal function tests) were conducted according to standard procedures by the clinical laboratory of The 2nd Xiangya Hospital. Additionally, six healthy blood samples were examined as controls.

The heart, liver, and kidney were removed immediately after animals were sacrificed, for pathological sectioning and HE staining to observe the organizational structure. Organ samples from six healthy rabbits were also examined as control tissues.

RESULTS

In vivo bio-safety evaluation

The blood biochemical test results are shown in Supplementary Figure 1. Results of blood biochemical tests (heart, liver and renal functions) showed that there are no significan differences between three groups in creatine kinase (CK), alanine aminotransferase (ALT) and Urea nitrogen (BUN) level, suggesting none of heart, liver or renal damage.

The organizational structures of heart, liver, and kidney were observed by pathological HE staining. No microscopic differences were noted in the tissue structures between three groups (Supplementary Figure 2).

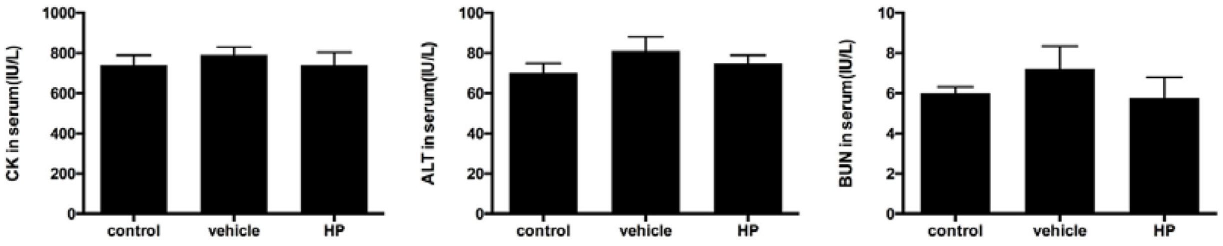

Supplementary Figure 1: Results of blood biochemical tests. Data represent the mean ± SD.Abbreviations: CK, creatine kinase; ALT, alanine aminotransferase; BUN, Urea nitrogen.

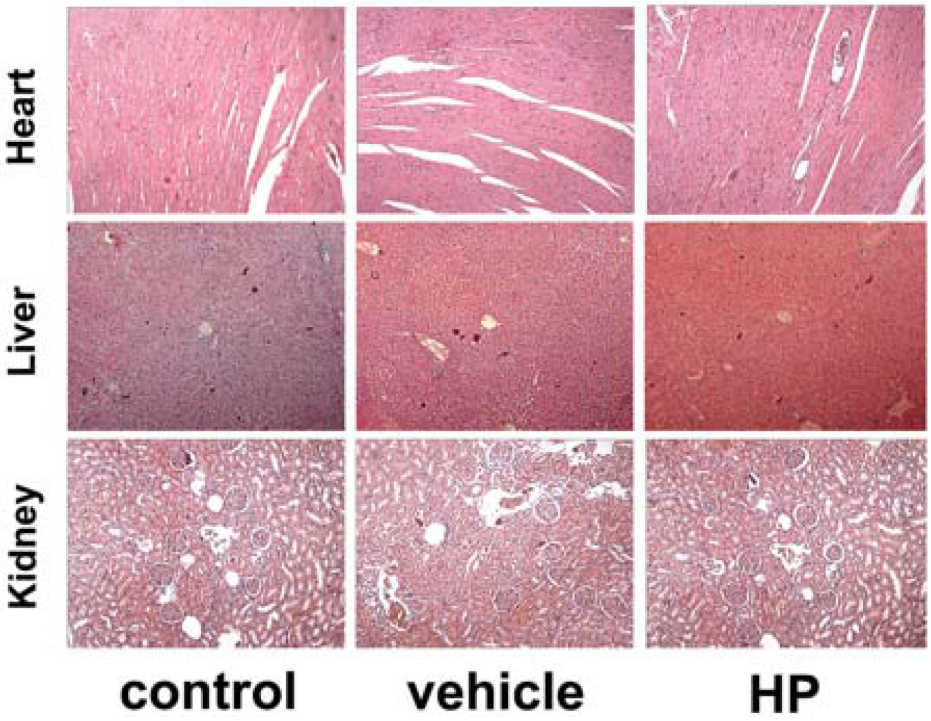

Supplementary Figure 2: Hematoxylin-eosin (HE) staining of longitudinal pathological sections of heart (400×), liver (100×), and kidney (100×) of the healthy controls and tumor bearing animals with/without application of HP.
